# Circadian misalignment on submarines and other non-24-h environments – from research to application

**DOI:** 10.1186/s40779-020-00268-2

**Published:** 2020-08-19

**Authors:** Jin-Hu Guo, Xiao-Hong Ma, Huan Ma, Yin Zhang, Zhi-Qiang Tian, Xin Wang, Yong-Cong Shao

**Affiliations:** 1 Key Laboratory of Gene Engineering of the Ministry of Education, State Key Laboratory of Biocontrol, School of Life Sciences, Sun Yat-sen University, Guangzhou, 510006 China; 2 China Institute of Marine Technology and Economy, Beijing, 100081 China; 3 School of Psychology, Beijing Sport University, Beijing, 100084 China

**Keywords:** Circadian rhythm, Circadian clock, Entrainment range, Metabolism, Alertness, Submarine

## Abstract

Circadian clocks have important physiological and behavioral functions in humans and other organisms, which enable organisms to anticipate and respond to periodic environmental changes. Disturbances in circadian rhythms impair sleep, metabolism, and behavior. People with jet lag, night workers and shift workers are vulnerable to circadian misalignment. In addition, non-24-h cycles influence circadian rhythms and cause misalignment and disorders in different species, since these periods are beyond the entrainment ranges. In certain special conditions, e.g., on submarines and commercial ships, non-24-h watch schedules are often employed, which have also been demonstrated to be deleterious to circadian rhythms. Personnel working under such conditions suffer from circadian misalignment with their on-watch hours, leading to increased health risks and decreased cognitive performance. In this review, we summarize the research progress and knowledge concerning circadian rhythms on submarines and other environments in which non-24-h watch schedules are employed.

## Background

Most organisms living on this planet possess circadian clocks that orchestrate their daily physiological and behavioral rhythms to the 24-h rotation period of the earth [1]. However, under special conditions in which the cycling periods do not extend over 24 h, the circadian systems may be challenged. In the laboratory, manual non-24-h conditions are established to study the basic features of circadian rhythms, although such conditions are very rare in nature. However, in human societies, a substantial group of people lives or works under non-24-h schedules. The non-24-h conditions were observed to lead to a decrease in adaptability to the environment in all tested organisms across different kingdoms, including bacteria, fungi, plants, and animals.

The circadian clocks are well conserved across species; thus, we will first introduce the knowledge derived from circadian studies in model organisms and the next focus on summarizing studies in humans. The former part would help to elucidate circadian misalignment in circadian rhythms, metabolism, sleep and the cognitive and behavioral consequences.

## Circadian clock and circadian rhythms

Circadian clocks govern life processes at multiple layers, including the molecular, cellular, organism and integral levels. At the organism level, circadian clocks are hierarchically composed of master clocks located in the pacemakers and peripheral clocks located in other tissues. In mammals, the pacemaker is the suprachiasmatic nucleus (SCN) in the anterior of the hypothalamus. In birds and reptiles, the eyes, pineal gland, or SCN are circadian pacemakers, depending on the species [1–3]. The peripheral clocks have their own circadian rhythms, but they can also be synchronized by pacemakers through the nervous and endocrine pathways [4].

**Fig. 1:**
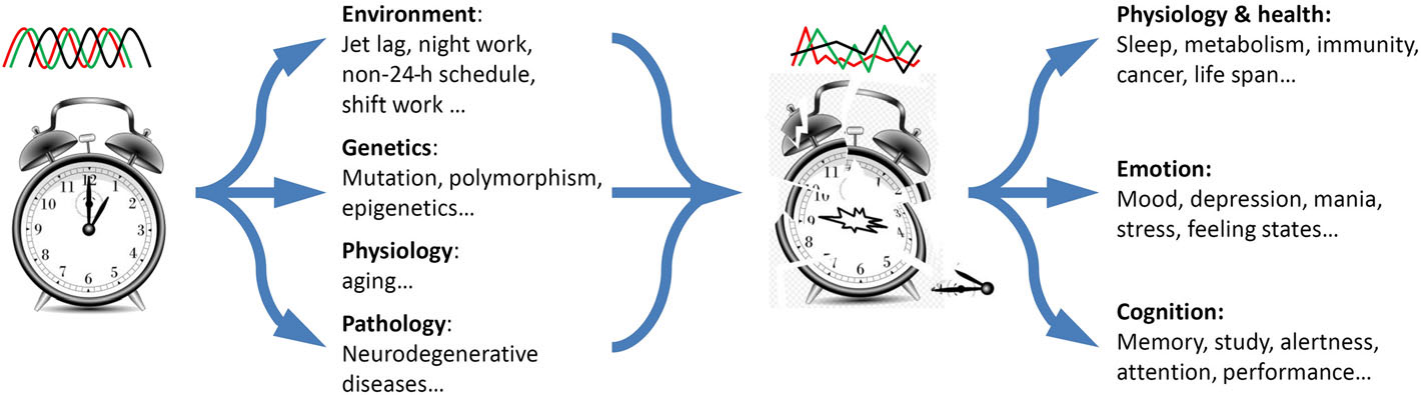
Circadian rhythm disturbance and their consequences

At the molecular level, circadian rhythms are generated by a set of core circadian genes, which constitute transcription-translational negative feedback loops [5]. Despite differences in circadian clock genes, the regulatory mechanisms are highly conserved across kingdoms [5, 6]. In mammals, including humans, positive circadian elements include brain and muscle Arnt-Like protein 1 (BMAL1)/2 and CLOCK, and the negative elements include PERIOD (PER) 1–3 and CRYPTOCHROME (CRY) 1/2 [7]. REV-ERBs (−α and -β) and retinoic acid receptor (RAR)-related orphan receptor (ROR) (−α, −β, and -γ) are additional regulators in mammalian circadian circuits, which act as ancillary loops to regulate *Bmail1* expression. REV-ERB proteins inhibit, while ROR proteins activate, the transcription of *Bmal1* [5, 7]. At the transcriptomic level, circadian clocks regulate the expression of approximately 40–80% of protein-encoding genes in mice [8, 9]. Via the output pathways, circadian clocks control most of the physiological and behavioral processes [10]. In mammals, circadian clocks govern the circadian/diurnal rhythms in sleep/wake cycles, feeding/fasting control, metabolism, hormone secretion, and immunity function (Fig. 1).

Although circadian rhythms are endogenous, they are subject to the influence of environmental, genetic, physiological and pathological factors (Fig. 1) [11, 12]. It has been demonstrated that disruptions in circadian rhythms impair adaptability in a variety of organisms [13]. In humans, compromised circadian rhythms cause chronic health consequences, including an increased risk of gastrointestinal illness, loss of bone mineral density, coronary artery disease, endocrine disruption, metabolic syndrome, and cancer. Moreover, disrupted circadian rhythms also affect emotions, cognition and performance (Fig. 1) [14–18].

## Entrainment range of circadian rhythms

The cycling periods of the environment, such as light and temperature, are defined as *T*-cycles. In a certain range, circadian rhythms can be adjusted to match the environmental periods, a process that is termed entrainment or synchronization. Circadian rhythms are both robust and flexible. Within a certain range, *T*-cycles that deviate from the natural 24-h cycles can also induce rhythms with the same periodicity as the environment. In this scenario, the endogenous circadian periodicity may be masked by the non-24-h *T*-cycles. The factors that can entrain circadian rhythms are known as zeitgebers, synchronizers or time givers [2, 19].

To experimentally determine the entrainment ranges, organisms are exposed to a series of fixed *T*-cycles or gradually changing *T*-cycles [20]. In addition, the entrainment range to an environmental cue can be inferred by the phase-response curve (PRC) information of the species. Given that according to the PRC of a species, the maximum phase delay and maximum phase advance are X hours and Y hours, respectively, the entrainment range of this species to the indicated cue would range from (24 h – Y) to (24 h + X) hours [1]. PRC represents the nonparametric effects of zeitgebers on entrainment. However, parametric components are also involved in determining the entrainment range. The parametric model assumes that light changes the velocity of the circadian rhythms for entrainment, while the nonparametric model emphasizes the phase shift [21].

**Fig. 2:**
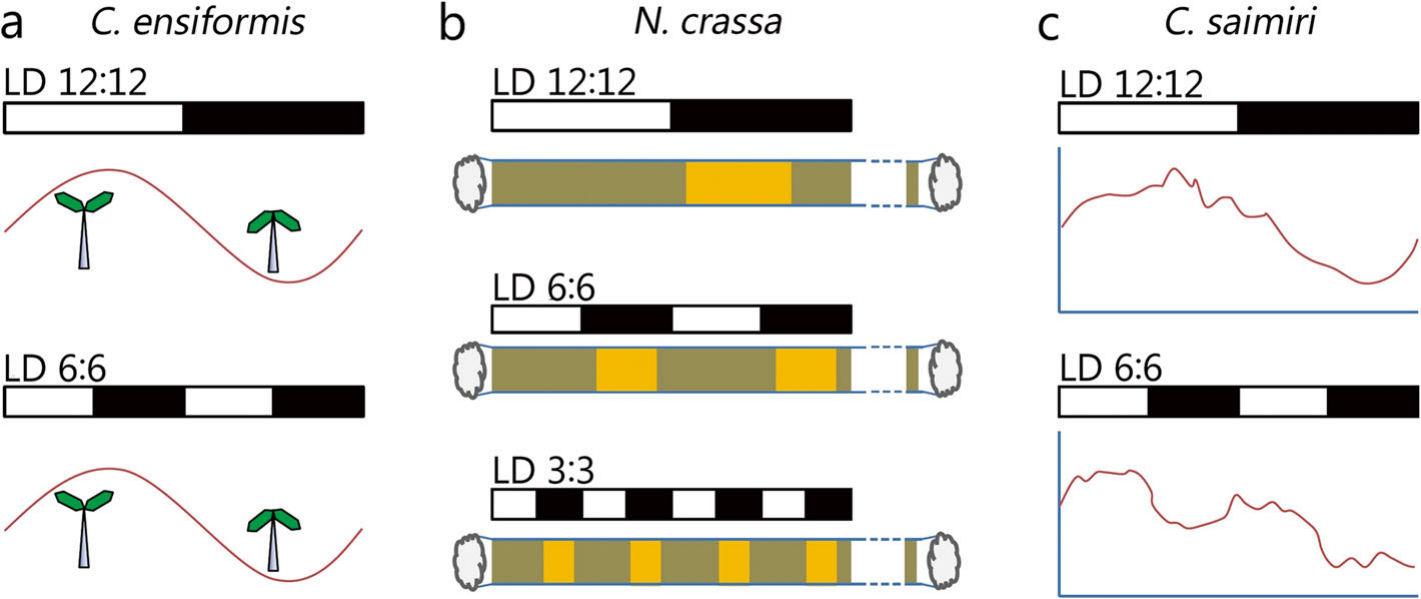
Circadian rhythms of different organisms in non-24-h conditions. **a** leaf movement (alternatively opening and closing) rhythms of *Canavalia ensiformis (C. ensiformis)*; **b** Conidiation banding rhythms of *N. crassa*. *Neurospora* is inoculated in a long and hollow glass tube with medium inside which is called a race tube. In the race tube, *Neurospora* grows towards the other end and releases conidiation bands colored orange. The release of conidiation is controlled by the circadian clock. Only the left part of the race tubes is shown; **c** rectal temperature rhythms of *C. Saimiri*. *Neurospora* conidiation rhythms are entrained in LD 12:12, LD 6:6 and LD 3:3, while *Canavalia* leaf movement rhythms and submarine crew rectal temperature rhythms were not entrained in LD 6:6. Therefore, they have different entrainment ranges. Panel **a** was drawn according to the study by [24], panel **b** was drawn according to the study by [25], and panel **c** was drawn according to the study by [26]

The entrainment ranges differ between species. Humans have a circadian clock with an endogenous period (*τ*) of approximately 25 h [22, 23] with upper and lower limits of ~ 22 and ~ 28 h, respectively [2]. In contrast, jack bean (*Canavalia ensiformis*) displayed leaf movement rhythmicity synchronized to light/dark (LD) 8:8 but not LD 6:6, suggesting that the lower entrainment limit is between 12 and 16 h [24] (Fig. 2a). Some species, such as bakery mold (*Neurospora crassa*) and house sparrow (*Passer domesticus*), show very wide entrainment ranges in banding rhythms of conidia release (conidiation) (Fig. 2b) and locomotor rhythms, respectively [2, 25, 27].

Circadian rhythms run free when the T cycles are beyond the entrainment ranges. Under LD 9:9, squirrel monkeys (*Cebidae Saimiri*) display a core body temperature (CBT) rhythm with a period of ~ 24 h instead of 18 h, suggesting the occurrence of a nearly free-running CBT rhythm (Fig. 2c) [26]. An approach has been proposed to measure the free-running period based on the displayed periods under non-24-h cycles beyond the entrainment ranges [28, 29]. Importantly, the rhythms beyond the entrainment ranges should be called superposition (or superimposition) [1, 24, 25]. Superposition may result in a period different from the one measured in a constant condition [20].

Despite the different entrainment ranges, different organisms exhibit rhythms with endogenous periods after transfer to constant conditions [24], suggesting the endogenous characteristics of the sustainability of circadian rhythms. In a study, the pregnant mice were kept in LD 28 or LD 20, and their progenies were also raised in these *T-*cycles until they reached puberty. The progenies showed free-running periods soon after they were placed in constant conditions [30]. The sole recorded exception was that hydrodictyon maintains a period of ~ 17.5 h in the constant dark for at least 3 days after having been retrieved from the LD 10.5:7 condition [24].

Different physiological and behavioral rhythms may also have different entrainment ranges within one species or even within individuals [20], suggesting the existence of independent oscillators. In addition to affecting the pacemaker of the circadian clock, zeitgebers also bypass it, directly impacting the overt rhythms, which causes internal desynchronization between different physiological processes as a consequence when *T* cycles are out of the entrainment ranges, e.g., the sleep-wake cycles and the CBT.

The molecular mechanisms determining the entrainment range have not been elucidated. The only clue regarding this mechanism was that in mice, *Clock* mutants exhibit a wider entrainment range (LD 24 to LD 32) compared to wild-type mice, suggesting that circadian clock genes play critical roles in regulating entrainment [31]. Mutants with less robust rhythms may be more prone to environmental entrainment, although such passive rhythms have no anticipating property, unlike circadian rhythms.

## Compromised adaptive fitness in non-24-h cycling conditions

The circadian clocks derive from the past long-term evolution, which confers the fitness of the cycling environment [32], and it is plausible that lives on earth cannot adapt to non-24-h cycling conditions in a short period of time. Two strategies were employed to test this hypothesis. One strategy is to study the effects of abnormal cycles of environmental cues on organisms, and the other strategy is to compare the competitiveness of mutants with different endogenous circadian period lengths or with no circadian rhythmicity. The adaptive fitness of a number of model organisms has been investigated concerning the circadian clock for the environment (Table 1).

**Table 1:** Selected studies changes in circadian rhythms and adaptation of model organisms under non-24-h conditions

Species	Non-24-h periods (h)	Adaptability	References
Cyanobacteria (*Synechococcus elongates*)	LD 11:11, LD 15:15	Decreased growth competence and adaptability	[33, 34]
Bakery mold (*Neurospora crassa*)	Temperature cycles: T12	Conidiation rhythms ran free	[35]
	LD 14:14, LD 9:9, LD 6:6	Conidiation rhythms were entrained	[25]
	LD 6, LD 18, LD 48	Conidiation rhythms were entrained	[27]
Tomato	LD 6:6, LD 24:24	Significantly decreased growth	[36]
Thale cress (*Arabidopsis thaliana*)	LD 10:10, LD 14:14	Decreased leaf chlorophyll content; decreased photosynthetic carbon fixation; decreased vegetative growth and survival	[37]
	LD 10:10, LD 14:14	The mutants showed a strong positive correlation in competition under the *T*-cycle growth conditions	[38]
Jack bean (*Canavalia ensiformis*)	LD 6:6, LD 8:8, LD 24:24	Leaf movement rhythms were entrained under LD 8:8, ran free under LD 6:6 and LD 24:24	[24]
Fruit fly (*Drosophila melanogaster*)	LD 21, LD 27	Reduced life span	[39]
Blowfly (*Phormia terraenovae*)	T26 T28	Decreased life spans under T26 and T28	[40]
House sparrow (*Passer domesticus*)	LD 6:6, LD 3:3, LD 1.5:1.5	Locomotion rhythms were at least partially entrained or showed superposition	[2]
Common chaffinch (*Fringilla coelebs*)	LD 22, LD 23, LD 25, LD 26	Activity rhythms were entrained to ranges from LD 23 to LD 25	[35]
Mouse (*Mus musculus*)	Temperature cycles: T20, 28	Superposition of luciferase rhythms in lung and SCN tissues, partially entrained	[41]
Nile grass rat *Arvicanthis niloticus*	T22–26	Locomotion rhythms were entrained between T = 23 and T = 25	[42]
Rat (*Rattus rattus*)	Feeding cycles: T20, T28	Decreased food intake and weight gain	[43]
Squirrel monkey (*Cebidae Saimiri*)	LD 9:9, LD 2:2	Core body temperature ran free, lower amplitude	[26]

Studies under asymmetric *T*-cycles are not included

*L* Denotes “light”, *D* denotes “dark”, *LD X:Y* Denotes cycling conditions with light for X hours and dark for Y hours alternatively, *SCN* Suprachiasmatic nucleus

All the available evidence suggests that living under circumstances with cycling periods beyond the entrainment ranges is harmful to reproductive fitness. Cyanobacteria strains possessing different endogenous circadian periods were mixed, and after a month in culture, the strain with a period resonating with the environment prevailed, while the strain with a period different from the environment failed in the competition [33, 34, 38]. Several models have been proposed to explain the clock-controlled fitness in Cyanobacteria, including the limited resource model, diffusible factor model cell-to-cell communication model, and the escape-from-light hypothesis [44], which may also help to characterize the enhanced fitness by the circadian clock in other organisms [37, 45]. The escape-from-light hypothesis postulates that in the early stage of evolution, the circadian clock acts to segregate light-sensitive reactions temporally to nighttime, e.g., cell division and DNA damage repair, to avoid the deleterious effects of radiation and oxidation from sunlight [37, 45].

Growth and flowing time were extensively affected, although the results varied in different species. A comparison of the growth of tomato under different LD conditions demonstrated that tomato grows most quickly in LD 12:12 but very slowly in either LD 24:24 or LD 6:6 [36]. Similar experiments have been conducted in a variety of species, including house sparrow, jack bean (*Canavalia ensiormis*), *N. crassa*, and squirrel monkey (*Saimiri sciureus*), which indicate that light regimens with periods that deviated dramatically from 24 h hampered growth or survival [2, 26, 46]. In a previous study, several plants were tested in a series of LD cycles, ranging from LD 5 s:5 s to LD 12:12; for instance, soybean (*Soja max* (*L.*) *IMper*) under LD 1 min:1 min showed a markedly chlorotic phenotype and a notable reduction in size, contained numerous spots of dead tissue, and showed a tendency to die prematurely [47]. Of course, the day length also contributes to the comprehensive influences on plants.

It has been demonstrated that in many organisms, non-24-h cycles have extensive negative or deleterious effects on survival and competitive fitness. Lesion of the SCN in antelope ground squirrels (*Ammospermophilus leucurus*) led to the loss of circadian rhythmicity in locomotor activity and an increased ratio of loss caused by predation [48]. The *tau* mutation in the enzyme casein kinase 1ε (CK1ε) had a dramatic influence on circadian periods in mice, with the heterozygous mice showing a two-hour shorter period, while the homozygous mice showed a four-hour shorter period. In the semi-natural environment, the relative frequency of the *tau* allele decreased to 20% from the initial half in 14 months due to disadvantages in both adult survival and the recruitment of juveniles into the cohorts [49].

In addition to fitness, misalignment of circadian rhythms with non-24-h cycling conditions impairs many physiological and metabolic processes. Moreover, the disruption of circadian rhythms leads to abnormalities in cognition in animals. The circadian control of cognition and behavior has been demonstrated in many animal species. Disruption of mouse circadian rhythms by LD cycles led to extensive ramifications in metabolism, brain function and behavior [50]. For instance, in LD 10:10, mice exhibited decreased cognitive flexibility and changes in emotionality, which were consistent with changes in neural architecture, including shorter dendritic length and reduced complexity of neurons in the medial prefrontal cortex [51].

In addition, non-24-h cycles shorten life spans in many tested organisms. For instance, Pittendrigh and Minis raised *Drosophila* under the conditions of *T* = 21 h and *T* = 27 h, and the life spans of *Drosophila* in both conditions were considerably shorter than those lived in *T* = 24 h [39].

A hypothesis proposed that when the cycling zeitgeber period is close to a demultiplicative period of the free-running period, the oscillator may also be entrained, which is known as frequency demultiplication [52]. However, frequency demultiplication is more likely to represent a passive response to cycling environmental stimuli than circadian oscillations [2, 24, 36, 52, 53].

## Effects of non-24-h conditions on human health and performance

### Non-24-h regimens affect circadian rhythms and sleep

Kleitman and Richardson were the first persons who recorded their experiences under a 28-h condition in a cavern of Mammoth National Park in Kentucky, USA. In the constant dark of Mammoth Cave, the sleep/wake cycles and body temperature of Richardson adapted to the 28-h cycles, but Kleitman failed [54]. In forced T21 and T27 schedules simulated in Spitzbergen, the body temperatures in 11 of 12 subjects adapted immediately, while the excretory rhythms adapted in only three subjects, suggesting the occurrence of desynchronization [55, 56]. Even periods with a slight difference from 24 h can be harmful to the growth or fitness of an individual. For instance, a study of the sleep-wake rhythms and sleep time in Mars Lander Phoenix, in which participants lived and worked on a 24.65-h schedule, which was in accordance with the daily cycling period of Mars, revealed inadaptation in the sleep-wake rhythms in some of the subjects and loss of sleep in almost all of the subjects [57].

Since the thirteenth century, the 4-h on/8-h off watch schedule has been employed in maritime crews, which is dramatically different from a 24-h day. In the military, the watch schedule of the US board vessel crews shifted in the last century from the 4-h on/8-h off routine to the 6-h on/12-h off routine to accommodate the three shift sections [58, 59]. Approximately 75% of enlisted men serving aboard U.S. Navy submarines stand watch on the latter schedule. In contrast, most officers and some senior enlisted men stand watch on a four-section rotation, i.e., 6 h on and 18 h off [60].

In studies of the circadian rhythms of submarine crew members, some physiological variables, including body temperature, serum or urine melatonin, cortisol and urine catecholamine, have been used as hallmarks [61–63]. In a 4-h on/8-h off schedule, the oral temperature of the subjects exhibited an ~ 24-h period, instead of a 12-h period [61]. Kelly et al. [63] investigated circadian rhythms of salivary melatonin in 20 crew members during a prolonged voyage on a Trident nuclear submarine and found that under 18-h duty cycles, the endogenous melatonin rhythm of the crew members showed an average period of 24.35 h.

In non-24-h cycles, the circadian parameters, including period, amplitude and phase, are all subject to change. During a prolonged voyage under a 4-h on/8-h off schedule, altered circadian rhythms in CBT occurred among the watchers. The most common phenomenon was the dampened amplitude. One subject even showed a free-running pattern [64]. Naitoh et al. [65] found that a 6-h on/12-h off routine during a nuclear submarine patrol caused a loss of 24-h rhythmicity in oral temperature, which was due not only to a decreased circadian amplitude but also to a dispersion of the time of the peak. A different watch time in three shift groups who used alternative 4-h on/8-h off regimens induced changes in the phase of rectal temperature, noradrenaline excretion, and adrenaline excretion [66].

Changes in circadian variables suggest that under non-24-h schedules, circadian rhythms are prone to alteration, and desynchronization may occur as a consequence. In 1979, Schaefer et al. analyzed the rhythms of body temperature, pulse rate and respiration rate of 11 crew members under two different schedules: an 18-h watch schedule (6-h on/12-h off) and a 24-h schedule [67]. The results showed that crew members using the former schedule but not the latter one exhibit superposition of 18-h and 24-h periods in all tested variables. Asimilar superposition was also observed in other studies [61, 62, 66]. As the schedule continues, the impact on circadian rhythms becomes more pronounced. Naitoh et al. [65] revealed that in a long-term voyage, the group synchronization in oral temperature rhythmicity disappeared after approximately 54 days.

Early in 1969, it was noticed that sleep insufficiency was caused by a 6-h on/12-h off schedule in the submarine crew [58]. In 1979, Schaefer et al. [67] measured the parameters of CBT, heart rate and respiration rate every 4 h for subjects living under different schedules, some of whom employed a 6-h on/12-h off the roster. Other subjects employed the normal 24-h schedule. The former schedule caused superposition on the rhythms, showing both 24-h and 18-h periods. Sleep insufficiency was also recorded in these subjects. A survey of 122 submarine crew members indicated that over 77% of them had medical complaints, and most complaints were about sleeping difficulty [68]. However, prevalent studies have demonstrated that under non-24-h conditions, both the quantity and quality of sleep are altered [67, 69, 70]. In real missions, the sleep time of the submarine crew varies in a wide range, extending from 5.75 h to 9 h, which may be influenced by many environmental factors [60].

Almost all studies indicated that non-24-h schedules elicit misalignment with the environmental cycles and deficiency in sleep quantity due to split sleep patterns, which means that sleep is separated into two or more parts during a day. Usually, both sleep quantity and sleep quality at night are better than those in the daytime [60, 69–73]. In contrast, Kosmadopoulos et al. [74] reported that a split sleep pattern showed no detrimental effects on sleep quality and performance, suggesting the necessity of conducting more extensive and systematic analyses.

Melatonin regulates sleep in a circadian fashion. For subjects living in an 18-h routine or a combined routine of 18 h and 24 h, the melatonin levels of these subjects showed periods between 24 h and 25 h, suggesting that free running occurred [63]. In contrast to the sleep-wake cycle, endogenous physiological parameters, including melatonin and temperature, have considerably narrower entrainment ranges. This discrepancy may cause desynchronization in physiology, psychology and behavior at the systemic level [65].

**Fig. 3:**
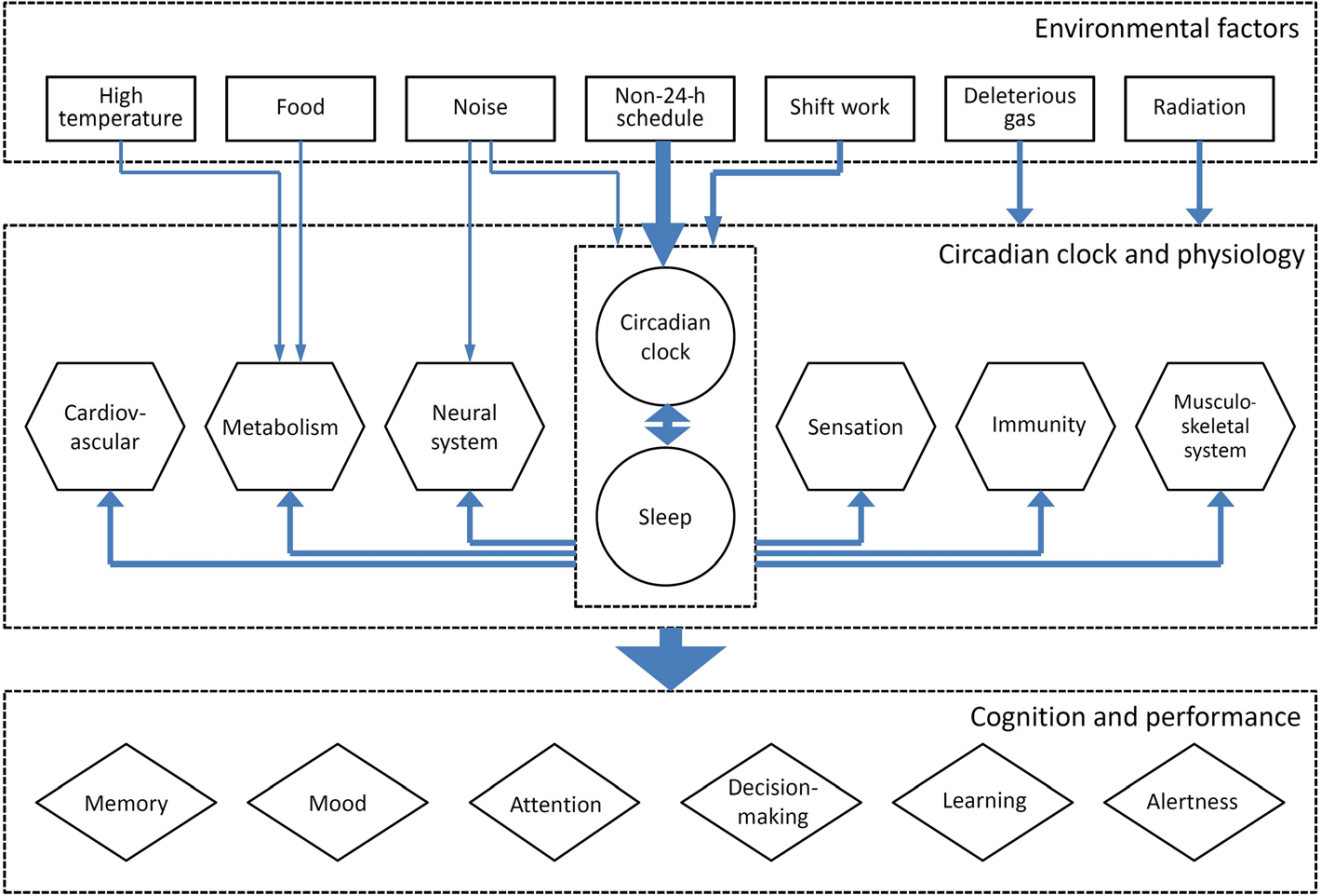
Disturbed circadian clock and impaired physiology and behavior in the submarine crew. Some connections between the components are not shown

In submarines, apart from non-24-h adherence to a rotating watch roster, other physical and chemical environmental cues in the cabin also influence the physiology and behavior of the crew members. These cues include confinement, deleterious atmospheres, sunlight deprivation and insufficient lighting, hypercarbia, noise, electromagnetic radiation, ionizing radiation, light spectrum and intensity, high temperature and humidity (Fig. 3) [59, 60, 63, 75–77]. The effects of many of these cues on human circadian rhythms have not been fully elucidated.

Moreover, in a real mission, jet lag may further contribute to the effects of circadian desynchronization. Condon et al. [66] found that the sleep length of watchkeepers was shorter when they crossed four to five time zones eastbound compared to crossing the time zones westbound, and the subjective alertness showed slower adaptation on eastbound travel. This finding may be explained by the fact that the human free-running period is ~ 25 h, which facilitates westbound adaptation. A 3-day shift regimen is widely employed in submariners [78], which may cause frequent shifts, such as social jet lag.

### Non-24-h regimens lead to changes in metabolism

The circadian clock governs metabolism by modulating the expression and function of essential genes within several important metabolic pathways in a temporal fashion. Conversely, misalignment in circadian systems disturbs metabolic homeostasis [7, 79]. The homeostasis of metabolism is also tightly associated with sleep [67, 68]. In mice, decreased food intake and weight were observed in mice raised under LD 20 or LD 28, suggesting that non-24-h cycling conditions may influence metabolic homeostasis [26]. In an anchored-sleep study, subjects were exposed to a schedule that enabled them to sleep for 4 h fixed from 0:00 to 4:00 and another 4 h unfixed during the day. The subjects showed altered acrophases in body temperature and urinary variables, including potassium and sodium [80], suggesting that their metabolisms were affected.

The level of insulin is under the control of the circadian clock, which peaks at night in humans [78]. Ma et al. [70] found that saliva insulin dramatically increased late at night and early in the morning in subjects under a schedule of 8 h for work and rest and 4 h for sleep. Consistently, the insulin levels and insulin: glucose ratios were increased in mice under LD 10 conditions [81]. Metabolic syndrome is a constellation associated with metabolic risk, including abdominal obesity, abnormal blood lipid levels, hypertension, and impaired fasting glucose. Kang and Song [82] reported that 513 submarine crew members had higher risks of metabolic syndrome and changed levels of low high-density lipoprotein cholesterol and impaired fasting glucose under a non-24-h on-watch schedule. Taken together, these data suggest that metabolism may be disturbed, which might be largely attributed to the non-24-h schedules.

In recent years, the involvement of the gut microbiome in animal and human metabolism has been increasingly recognized. Changes in the category and the abundance of gut and oral microbiota also display a circadian pattern [83–86]. Changes in alimentary tract microbiota account for alterations in host metabolism. Relative to the fecal microbiome, the salivary microbiome has rarely been studied, despite its higher accessibility. The oral microbiome contains bacteria, viruses and fungi, which associate and form biofilms. Environmental changes might convert them to be pathogenic and trigger intestinal diseases or other inflammatory diseases [87]. Recently, Ma et al. [70] found that disruption of the composition of the oral microbiome occurred in subjects living under a non-24-h schedule. Some bacterial species showed specific change patterns, e.g., Tenericutes and SR1, corresponding to sleep deprivation and circadian desynchronization, respectively.

### Non-24-h regimens affect mood, cognition and performance

Fatigue is an important cause of accidents and incidents at sea [73]. One requirement of safe submarine operations is to ensure that sailors work at their highest level of readiness. The non-24-h on-watch schedules cause misalignment in circadian rhythms which, in turn, leads to sleep deprivation [63, 88]. Impairments in circadian rhythms and sleep have extensive physiological and cognitive effects, including increased fatigue and changes in mood, vigilance, and work productivity (Figs. 1 and 3). It is critical to pay close attention to the circadian rhythms and performance in submarine crews, as Moore-Ede pointed out: “There should be some global concern about the health and performance of these men, since they are the ones with their fingers directly on the nuclear button!” [26].

Naitoh et al. [65] assessed the mood changes in a group of nuclear submariners by using subjective questionnaires of Thayer’s activation, Mood “Activity” (MA) and Mood “Happiness” (MH) and revealed that during a 10-week submerged patrol, the 24-h rhythmicities of Mood “Activity” and Mood “Happiness” were abolished compared to the control period. In another study, the results from mood scale questionnaires also indicated that compared to the patrol period, significantly more feelings of “Activity” and “Happiness” were observed in the postpatrol period [60].

In humans, the circadian nadir (trough) of CBT and alertness is late at night and early in the morning, and changes in body temperature reflect the dynamics of metabolism, which is also one of the determinants of alertness [1, 89–91]. Under the 4-h on/8-h off schedule, the results of the Letter Cancellation Test and Vector Test from watchkeepers demonstrated in the case of the former that the “best” time is forenoon, while in the latter, it is late afternoon. The slowest performance occurred either at the start of watches beginning immediately after wakening or near 4:00 in the morning, which is the nadir of alertness [92]. Under submarine watch routines, both misalignments in circadian rhythms and decreases in sleep length and quality impose negative effects on mood, cognition and performance [72, 74]. During 2007, the effects of the existing watch schedules used in Canadian Forces were evaluated, revealing that the cognitive efficacy in the submarine crew dramatically decreased to a dangerously low level [93]. Ma et al. [70] showed that under a 4-h on/8-h off routine, the alertness of the subjects decreased dramatically in the morning. As chronic sleep deprivation may occur during non-24-h cycles, it is unclear whether misaligned circadian rhythms or sleep loss contributed more to the decreased alertness.

The Fatigue Avoidance Scheduling Tool (FAST™) program has been developed to optimize the scheduling and performance of the Air Force. Consideration and integration of more factors, such as individual differences, might help to improve the prediction accuracy and practicability in not only the Air Force but also for personnel working under conditions with non-24-h periods, including submarine crew members [94].

To date, many studies have documented the impairment of non-24-h schedules on circadian rhythms and cognition, and most of these studies have been conducted at the physiological level. By contrast, the molecular mechanisms governing the effects of disrupted circadian rhythms have not been elucidated, which may thus limit the in-depth understanding of the physiological changes and the development of countermeasures.

## Countermeasures, conclusions and perspectives

Circadian disruption profoundly affects health and performance in military and commercial vessel crews. During a 90-day voyage, which is the longest voyage recorded to date, disturbance in circadian rhythms was reported in many Chinese crew members. Most of the crew members experienced sleep disorders, flagging, dysphoria and faintness. Some crew members attempted to overcome these conditions by chewing dry peppers or daubing menthol camphorate on their temples [95]. In maritime missions, circadian misalignment causes serious consequences, including high personnel turnover (30–50%) per voyage and requirements to train the new entrant [26].

However, the question remains of how we can overcome circadian misalignment and avoid deleterious consequences. The entrainment range needs to be considered. If the environmental period is within or close to the entrainment range, it may be useful to use countermeasures to adjust the circadian rhythms. If the environmental period is far beyond the entrainment range, it may be useful to construct a man-made 24-h-period microenvironment. It is also possible to combine both approaches. Some efforts have been made to lessen the deleterious effects of disrupted circadian rhythms and to improve health and performance, which primarily involved optimization of the watch schedules and modifications of the zeitgebers.

Optimized watch schedules may improve circadian rhythms and performance. Duplessis et al. [59] compared the impacts of different watch schedules (6-h on/8-h off and a compressed close-6 h watch schedule), and the wrist activity results showed that sleep duration was longer under the 6-h on/8-h off schedule. Sleep discontinuity appears to be considerably less severe on the 6-h on/12-h off watch schedule than that of the 4-h on/8-h off watch schedule. In contrast, the 6-h on/12-h off schedule allowed more continuous sleep [60], which might be explained by the fact that T12 is shorter than *T*18 compared to human FRP. The best approach is to use a 24-h roster instead of a non-24-h roster. A 24-h schedule was used during an operational tour broad the USS MARYLAND, a U.S. Navy ballistic missile submarine, and positive feedback regarding fatigue, energy level, sleep inertia, and morale were obtained from the crew [58]. In 2014, the US Navy ordered all submarines to change to a 24-h schedule by December 2014 [96, 97]. It is believed that the 24-h schedule works better to improve mood and prompt social interaction. However, to this end, the prerequisite will be that the work environments have sufficient accommodability for alternate teams.

Even under the same regimens, the adaptation varies among shift sections. Under 4-h on/8-h off schedules, the three watch groups showed different cognitive patterns, which suggests different degrees of adaptation [93]. The interval between shifts is also a determinant of the performance [98]. If shifts change too frequently, the circadian rhythms of the subjects will lose synchrony, similar to the effects of jet lag. If shifts change less often, some of the teams will work at a ‘low’ status, similar to the night workers. In a long-term voyage, inner cues, such as lights and mealtime, might act as weak daily cycling zeitgebers in the cabin without exposure to natural light. In this scenario, there are always some personnel working at artificial nighttime.

Individual variation in the flexibility of circadian rhythms has been reported in several studies [62, 65]. In fact, screening and employing personnel who show higher tolerance to non-24-h cycles or shifts has been suggested. However, although some people show adaptation in sleep/wake cycles [65], extensive modulations of other rhythms have been observed; thus, the potential desynchronization in their inner rhythms and its long-term impacts on health and performance need to be further evaluated.

Some commonly used medications, e.g., dextroamphetamine, caffeine and modafinil, can temporally preserve or restore alertness and vigilance as pharmacological countermeasures under sleep deficiency [99]. Some other hypnotics, e.g., nonbenzodiazepines and melatonin receptor agonists, can improve the quantity and quality of sleep with fewer side effects [100]. However, these medications are usually used on short-term missions instead of long-term missions [101]. Cognition-enhancing equipment, such as transcranial direct current stimulation (tDCS), is widely used, but there is little evidence supporting the anticipated effects of this equipment [102].

The strength of the zeitgeber, such as the light intensity, also affects the range of entrainment [20]. Blue or white light has been used for light therapy of circadian rhythm-associated disorders and enhancement of alertness and performance [103]. In the new 24-h watch schedule in submarines, highly correlated color temperature (CCT = 13,500 K) fluorescent light sources function better in promoting alertness and performance to be aligned with the environmental cues [97]. Light therapy can improve circadian rhythmicity and performance in short-term missions, which may result in further aggravation under long-term non-24-h schedules.

It has been demonstrated that exposure to gradually changing environmental cycles may enlarge the entrainment range, e.g., gradually increasing/decreasing LD periods and feeding periods [20]. LD twilight exposure led to wider entrainment ranges in hamsters or deer mice compared to rectangular LD cycles [21, 104]. Similarly, sinusoidal light-intensity cycles also broadened the entrainment limits in rats [105, 106]. The effects of enhancing the entrainment ranges by changing zeitgebers may be attributed to parametric mechanisms [21].

Overall, the influences of non-24-h environments on physiology and behavior warrant further study, both in basic research and applied research. Many factors need to be considered to optimize the performance. Similar non-24-h schedules are also widely implemented in maritime operations and industries in addition to submarines, including oil mining, merchant vessels and some other industries and air crews [93, 107], suggesting a substantial population size of affected subjects. In the future, space exploration will expose humans to non-24-h cycling environments. For orbital space crafts, the LD cycling period is approximately 90 min [108]. The self-rotation periods of the Moon and Mars are ~ 14 days and 24.65 h, respectively [109]. Some countries, including the US and China, are planning to send humans to the moon and Mars in the near future, demonstrating the importance of the adaptation of circadian rhythms in exotic environments.

## Box 1. Glossary of some key terms

**Table:** 

**Circadian:** a term derived from the Latin roots “circa” and “diem,” meaning “about a day”. **Circadian rhythm** refers to a self-sustaining periodic variation of molecular, physiological or behavioral events, with a cycle of approximately 24 h in constant condition. **The circadian clock** refers to the endogenous mechanisms generating circadian rhythms in different organisms.	
**Free-running period (FRP)**: the period of a circadian rhythm running under constant conditions, with no changes in the environmental cues (e.g., light, temperature, etc.).	
**Suprachiasmatic nucleus (SCN):** a small region of two bilateral clusters of nerve cells located above the optic chiasm in the anterior hypothalamus in mammalian brains. The SCN receives light stimulus via the eyes and is responsible for coordinating circadian rhythmicity in peripheral tissues.	
**Zeitgeber time (ZT):** Zeitgeber is a German word meaning “time giver”, which is an environmental cue (e.g., light or food) that entrains the circadian rhythms. Zeitgeber time refers to a standard of time based on the period of a zeitgeber, such as the 24-h diurnal cycle of light and darkness.	
**DD**: conventional notation for an environment maintained in continuous darkness, as it stands for dark-dark conditions without light exposure.	
**LD**: conventional notation for a light-dark environmental cycle. The quantity and ratio of time in light and dark can be presented separated by a colon. For instance, LD 12:6 denotes a cycle consisting of alternation of 12 h in light and 6 h in dark.	
**Entrainment:** adjustment of the period and phase of an internal circadian oscillator to environmental cues with regular intervals. It is also called synchronization or resetting.	
**Entrainment range:** circadian rhythms can enter non-24-h zeitgeber cycles (e.g., light-dark cycles, temperature cycles or cycling social cues) but only within given limits.	
**Nadir:** lowest value of a rhythmic function. Nadir is the synonym of trough.	
**Phase:** the instantaneous state of an oscillation relative to a reference point within a cycle.	
**Phase-response curve (PRC):** a curve describing the phase shifts caused by a stimulus, such as light exposure or pharmacological treatment, as a function of the phase (i.e., circadian time) at which the manipulation occurs.	
**Amplitude**: the magnitude from the mean level of rhythmic values to either the peak or nadir.	
**Period**: the duration of a complete cycle of circadian rhythms. Period is measured from specific phase points in between the intervals of neighboring cycles, for example, peak-to-peak or trough-to-trough.	
**Psychomotor vigilance task (PVT)**: a computer-based test to chronometrically measure the reaction of the subjects to specified sustained-attention and reaction-timed tasks. Operational alertness and accuracy can be inferred and analyzed based on the PVT results.	

## Data Availability

Not applicable.

## Abbreviations

BMAL1: Brain and muscle Arnt-like protein 1
CBT: Core body temperature
CRY: Cryptochrome
FRP: Free-running period
KSS: Karolinska Sleepiness Scale
IGF: Impaired fasting glucose
LD: Light/dark
PER: Period
PVT: Psychomotor vigilance task
ROR: Retinoic acid receptor-related orphan receptor
SCN: Suprachiasmatic nucleus
